# Expanding Outcomes Beyond Chronic Kidney Disease-Mineral and Bone Disorder: A Propensity Score-Matched Analysis of Parathyroidectomy versus Calcimimetics in Dialysis Patients with Secondary Hyperparathyroidism

**DOI:** 10.7150/ijms.131653

**Published:** 2026-05-22

**Authors:** Yi-Chou Hou, Cai-Mei Zheng, Ko-Lin Kuo, Kuo-Wang Tsai, Joshua Wang, Cheng-Yi Wang, Ruei-Ming Chen, Kuo-Cheng Lu

**Affiliations:** 1 Division of Nephrology, Department of Internal Medicine, Cardinal Tien Hospital, School of Medicine, Fu Jen Catholic University, New Taipei City, Taiwan.; 2 Division of Nephrology, Department of Internal Medicine, Shuang Ho Hospital, Taipei Medical University, New Taipei City, Taiwan.; 3 Division of Nephrology, Department of Internal Medicine, School of Medicine, College of Medicine, Taipei Medical University, Taipei, Taiwan.; 4 TMU Research Centre of Urology and Kidney, Taipei Medical University, Taipei, Taiwan.; 5 Division of Nephrology, Department of Medicine, Taipei Tzu Chi Hospital, Buddhist Tzu Chi Medical Foundation, New Taipei City, Taiwan.; 6 School of Medicine, Tzu Chi University, Hualien, Taiwan.; 7 School of Post-Baccalaureate Chinese Medicine, Tzu Chi University, Hualien, Taiwan.; 8 Department of Research, Taipei Tzu Chi Hospital, Buddhist Tzu Chi Medical Foundation, New Taipei City 23142, Taiwan.; 9 Department of Nursing, Cardinal Tien Junior College of Healthcare and Management, New Taipei City 23143, Taiwan.; 10 Division of Pulmonology, Department of Internal Medicine, Cardinal Tien Hospital, School of Medicine, Fu Jen Catholic University, New Taipei City, Taiwan.; 11 Cell Physiology and Molecular Image Research Center, Wan Fang Hospital, Taipei Medical University, Taipei, Taiwan.; 12 Graduate Institute of Medical Sciences, College of Medicine, Taipei Medical University, Taipei, Taiwan.; 13 Anesthesiology and Health Policy Research Center, Taipei Medical University Hospital, Taipei, Taiwan.; 14 Division of Nephrology, Department of Medicine, Fu Jen Catholic University Hospital, School of Medicine, Fu Jen Catholic University, New Taipei City, Taiwan.

**Keywords:** end-stage renal disease, secondary hyperparathyroidism, parathyroidectomy, calcimimetics, mortality, cognitive impairment, sepsis

## Abstract

**Background:**

Secondary hyperparathyroidism (SHPT) is a common complication in patients with end-stage renal disease (ESRD) receiving dialysis. Although calcimimetics (CAMs) and parathyroidectomy (PTx) are widely used for severe SHPT, their comparative effects on long-term clinical outcomes beyond mineral and bone disorder remain uncertain.

**Methods:**

We conducted a retrospective multicenter cohort study using the TriNetX research network (2010-2024). Adult dialysis patients with ESRD and severe SHPT, defined by at least one historical intact parathyroid hormone level >600 pg/mL prior to treatment initiation, were included. Patients receiving both therapies were excluded. After 1:1 propensity score matching for demographics, comorbidities, medications, and laboratory variables, outcomes including all-cause mortality, major adverse cardiovascular events (MACE), fracture, cognitive impairment, mild cognitive impairment (MCI), and sepsis were compared using Cox proportional hazards models.

**Results:**

After propensity score matching, 22,580 patients were included (11,290 in each group), with follow-up extending to 7 years. Compared with CAM therapy, PTx was associated with significantly lower risks of all-cause mortality (hazard ratio [HR] 0.914, 95% confidence interval [CI] 0.860-0.972), mild cognitive impairment (HR 0.646, 95% CI 0.436-0.955), and sepsis (HR 0.844, 95% CI 0.765-0.930). No significant differences were observed for MACE, fracture, hungry bone disease, overall cognitive impairment, or malignancy.

**Conclusion:**

In patients with ESRD and severe SHPT, parathyroidectomy was associated with lower risks of mortality, mild cognitive impairment, and sepsis compared with calcimimetic therapy, while cardiovascular and fracture outcomes were comparable. These findings suggest that PTx may confer broader systemic benefits beyond mineral and bone disorder control in selected dialysis patients.

## Introduction

Secondary hyperparathyroidism (SHPT) represents a frequent and debilitating complication in patients with end-stage renal disease (ESRD) undergoing dialysis. SHPT results from the interplay of phosphate retention, hypocalcemia, and reduced vitamin D activation, leading to chronic stimulation of the parathyroid glands 1, 2. Sustained hyperparathyroidism causes metabolic bone disorder, vascular calcification, anemia, and systemic inflammation. Elevated parathyroid hormone (PTH) levels are not only markers of mineral imbalance but also correlate with higher mortality, hospitalization, and impaired quality of life 3.

Therapeutic approaches for SHPT include pharmacological treatment with calcimimetics (CAMs) and surgical parathyroidectomy (PTx). CAMs such as cinacalcet and etelcalcetide allosterically activate the calcium-sensing receptor to reduce PTH secretion 4, 5. They have demonstrated efficacy in lowering serum PTH, calcium, and phosphorus. However, their long-term impact on hard clinical outcomes remains controversial, as randomized controlled trials (RCTs) have yielded mixed results 6, 7. PTx, by contrast, directly removes hyperplastic parathyroid glands, leading to sustained biochemical control and often greater improvements in calcium-phosphate homeostasis.

Despite these mechanistic differences, evidence comparing PTx and CAMs in real-world populations remains limited. Most prior studies have focused on mortality or cardiovascular outcomes, while neglecting broader systemic complications of ESRD 7, 8. Two outcomes of particular relevance are cognitive impairment and infection risk. Cognitive impairment is highly prevalent among dialysis patients, with estimates ranging from 30% to 60% 9, 10. It arises from a combination of uremic toxin accumulation, cerebral microvascular disease, oxidative stress, and chronic inflammation. Similarly, sepsis is one of the leading causes of death and hospitalization in dialysis patients, reflecting impaired immune function and frequent vascular access exposure 11. Whether improved biochemical control from PTx translates into reduced neurocognitive decline or lower sepsis burden has not been systematically studied.

The present study therefore addresses this critical gap. Using a large federated network of electronic health records, we compared the long-term outcomes of ESRD patients with severe SHPT treated with PTx versus CAMs. We hypothesized that, beyond survival, PTx would confer systemic benefits, including reduced risk of cognitive impairment and sepsis, compared with CAM therapy.

## Materials and Methods

Study Design and Data SourceThis was a retrospective multicenter cohort study conducted using the TriNetX research network, which integrates de-identified electronic medical records (EMRs) from 84 healthcare organizations. The database includes demographic characteristics, diagnoses, procedures, medications, laboratory values, and longitudinal outcomes. The study period spanned from January 1, 2010, to June 30, 2024, with a maximum follow-up duration of 7 years.

### Study Population and Index Date

This retrospective cohort study was conducted using the TriNetX research network. Eligible patients were adults with end-stage renal disease (ESRD) who subsequently developed severe secondary hyperparathyroidism (SHPT), defined as the presence of at least one documented intact parathyroid hormone (iPTH) measurement exceeding 600 pg/mL. ESRD was identified by the ICD-10-CM diagnosis code **N18.6** for 3 times.

Patients were classified into two mutually exclusive treatment groups:

- Parathyroidectomy (PTx) cohort: Patients who had undergone parathyroidectomy after initiating dialysis. PTx was identified by ICD-10-PCS and CPT procedure codes, including excision, resection, destruction, subtotal, or total parathyroidectomy procedures. The index date was defined as the date of the first qualifying parathyroidectomy (Table 1).
- Calcimimetic (CAMs) cohort: Patients who received calcimimetic therapy (cinacalcet, etelcalcetide, or evocalcet; coding definitions are listed in Table 1) after dialysis initiation, with iPTH >600 pg/mL at baseline. The index date was defined as the date of the first prescription of a calcimimetic agent.

Patients were excluded if they received both PTx and CAM therapy, either before the index date or within ±90 days of each other, to ensure mutually exclusive exposure groups. Patients with iPTH ≤600 pg/mL prior to treatment, those with acute kidney injury (AKI), or those lacking complete demographic or follow-up information were also excluded.

The analysis time window was defined from 365 days after the index event to 2555 days (7 years) after the index event. This approach minimized immortal time bias by excluding outcomes within the first year after treatment initiation and ensured sufficient follow-up for long-term outcomes. Patients whose index event occurred more than 20 years prior to the analysis date were excluded, although no such cases were identified in this study.

### Exposures and Outcomes

The primary and secondary study outcomes were defined using standardized ICD-10-CM codes listed as Table 1. All-cause mortality was determined based on death status recorded in the electronic health record. Major adverse cardiovascular events (MACE) were defined as a composite of ischemic heart diseases (I20-I25), cerebral infarction (I63), heart failure (I50), diseases of arteries, arterioles, and capillaries (I70-I79), and other cardiac arrhythmias (I49). Fracture events were identified using diagnostic codes for fractures of the cervical spine and neck (S12), thoracic spine and rib cage (S22), lumbar spine and pelvis (S32), shoulder and upper arm (S42), forearm (S52), wrist and hand (S62), femur (S72), lower leg including ankle (S82), and foot or toe excluding ankle (S92). Hungry bone syndrome was defined by either the presence of a diagnosis code for hungry bone syndrome (E83.81) or hypertrophy of bone of unspecified site (M89.30).

Neurocognitive outcomes were also included. Cognitive impairment and dementia encompassed unspecified dementia (F03), dementia in other diseases classified elsewhere (F02), Alzheimer's disease (G30), and vascular dementia (F01). In addition, mild cognitive impairment (MCI) was defined as the presence of a diagnosis code for mild cognitive impairment of uncertain or unknown etiology (G31.84). Malignancy was defined by incident diagnoses of malignant neoplasms involving the lip, oral cavity, and pharynx (C00-C14); digestive organs (C15-C26); respiratory and intrathoracic organs (C30-C39); breast (C50); and urinary tract (C64-C68). Finally, sepsis was defined as a diagnosis of sepsis (A41).

### Covariates

Baseline variables included demographics (age, sex, race/ethnicity), comorbidities (diabetes mellitus, hypertension, ischemic heart disease, heart failure, cerebrovascular disease, peripheral vascular disease, chronic obstructive pulmonary disease, chronic liver disease, malignancy), and dialysis vintage. Laboratory data, when available, included hemoglobin, serum calcium, phosphate, and estimated glomerular filtration rate (eGFR).

### Statistical Analysis

Propensity score matching was conducted on 32 baseline characteristics, including demographics (age, sex, race, ethnicity), comorbidities (hypertension, ischemic heart disease, cerebrovascular disease, diabetes, and other cardiovascular conditions), medication use (ACE inhibitors, angiotensin II inhibitors, vitamin D, calcium channel blockers, bisphosphonates, calcium, GLP-1 analogues, DPP-4 inhibitors, and other bone/mineralization agents), and laboratory parameters (intact parathyroid hormone, serum calcium, hemoglobin, and calcidiol). Cohorts were balanced 1:1 using these covariates, and the baseline characteristics before and after matching are summarized in Table 2. Kaplan-Meier survival curves were generated, and hazard ratios (HRs) with 95% confidence intervals (CIs) were estimated using Cox proportional hazards models. Subgroup analyses were performed to explore whether the association between treatment strategy and outcomes varied across predefined clinical and biochemical strata. Hazard ratios were calculated by comparing patients with versus without each specified factor. These analyses were intended to assess effect modification rather than to determine independent causal effects of individual variables. A two-sided p-value < 0.05 was considered statistically significant.

## Results

### Study Population

**Figure 1:**
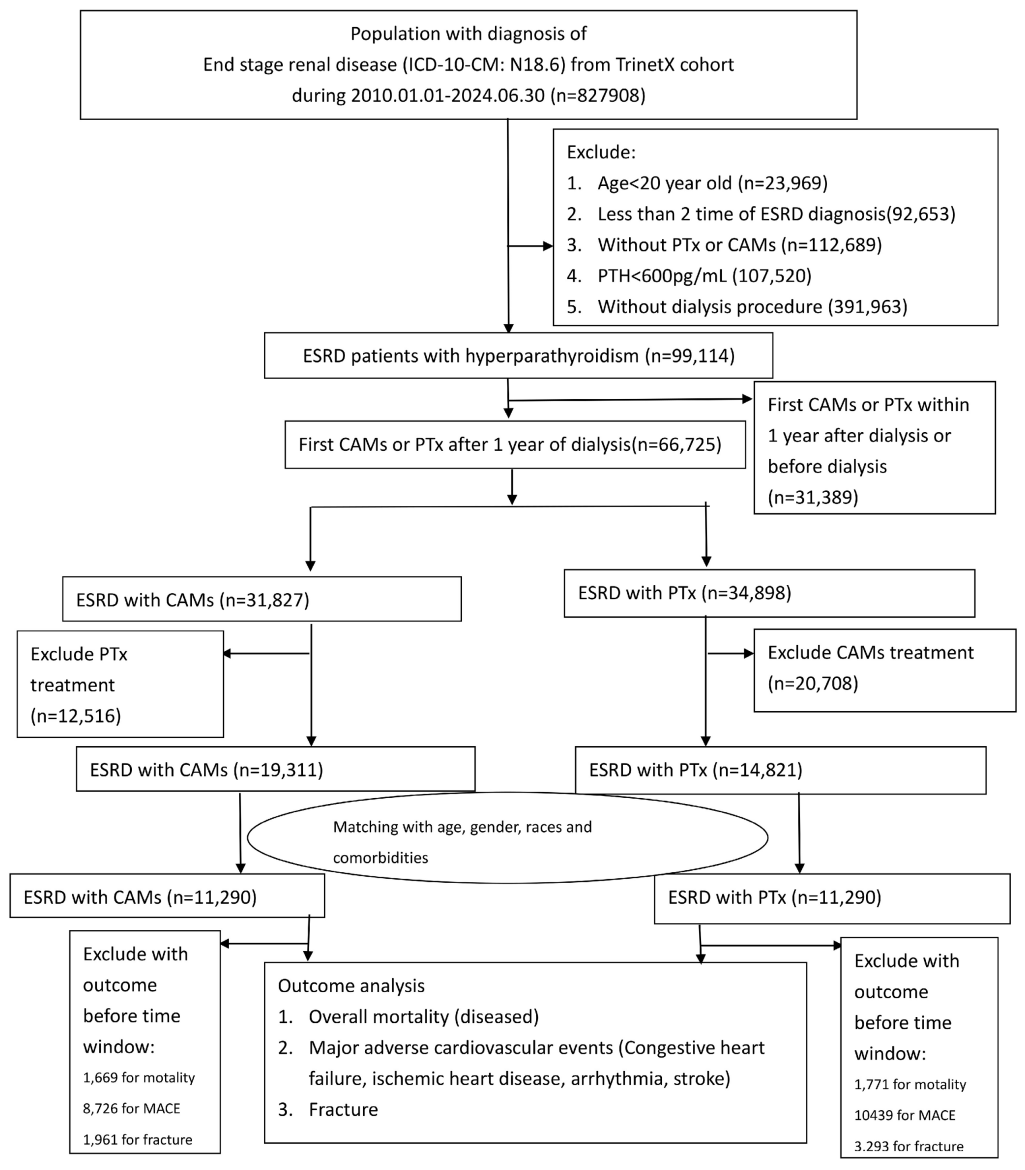
Flow chart of patient selection. Patients with end-stage renal disease (ESRD; ICD-10-CM: N18.6) were identified from the TriNetX research network (2010-2024). After applying sequential exclusions for age <20 years, insufficient ESRD diagnoses, absence of dialysis procedures, parathyroid hormone (PTH) <600 pg/mL, early treatment exposure, and prior outcomes, 11,290 patients undergoing parathyroidectomy (PTx) and 11,290 patients receiving calcimimetics (CAMs) were included following 1:1 propensity score matching for demographics, comorbidities, laboratory data, and medication use.

Figure 1 illustrated the flow chart of the study. A total of 34,133 patients with ESRD and severe SHPT (PTH > 600 pg/mL) were identified. After 1:1 propensity score matching, 22,580 patients remained in the analytic cohort, comprising 11,290 who underwent PTx and 11,290 treated with CAM therapy.

Before matching, the PTx and CAMs groups differed significantly in demographic, diagnostic, and medication profiles. After 1:1 propensity score matching, baseline characteristics were well balanced: age, sex, race, and ethnicity were comparable between groups; the prevalence of major comorbidities, including hypertension, diabetes, ischemic heart disease, and cerebrovascular disease, was similar; and the distribution of medication use (ACE inhibitors, angiotensin II inhibitors, vitamin D, calcium channel blockers, bisphosphonates, calcium, GLP-1 analogues, and DPP-4 inhibitors) was nearly identical. All standardized differences were reduced to < 0.1, confirming adequate covariate balance across matched cohorts (Table 2). The laboratory results were listed as Table 3.

### The Comparison between CAMs and PTx

**Figure 2:**
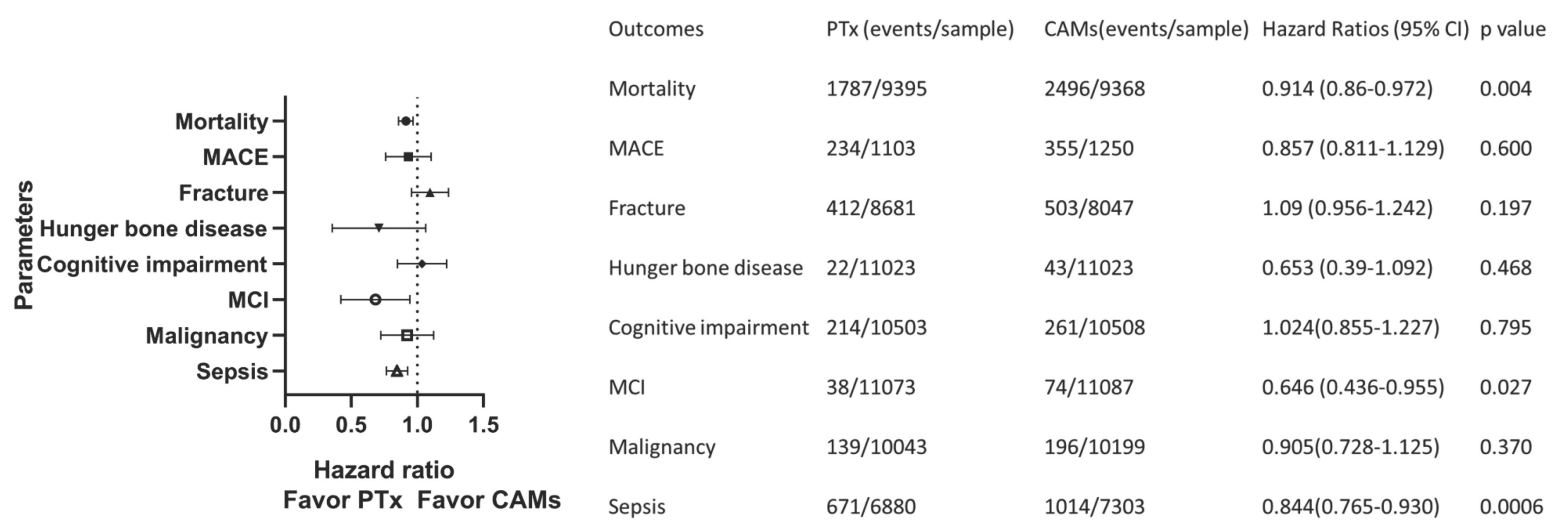
Forest plot of major outcomes comparing PTx and CAMs after propensity score matching. PTx was associated with lower risks of all-cause mortality (HR 0.914, 95% CI 0.860-0.972), mild cognitive impairment (HR 0.646, 95% CI 0.436-0.955), and sepsis (HR 0.844, 95% CI 0.765-0.930). No significant differences were observed for MACE, fracture, hungry bone disease, overall cognitive impairment, or malignancy.

Figure 2 shows the hazard ratios for major outcomes after propensity score matching. Compared with CAM therapy, PTx was associated with significantly lower risks of all-cause mortality (HR 0.914, 95% CI 0.860-0.972, p = 0.004), mild cognitive impairment (HR 0.646, 95% CI 0.436-0.955, p = 0.027), and sepsis (HR 0.844, 95% CI 0.765-0.930, p = 0.0006). No significant differences were observed for MACE, fracture, hungry bone disease, overall cognitive impairment, or malignancy.

### All-cause Mortality

**Figure 3:**
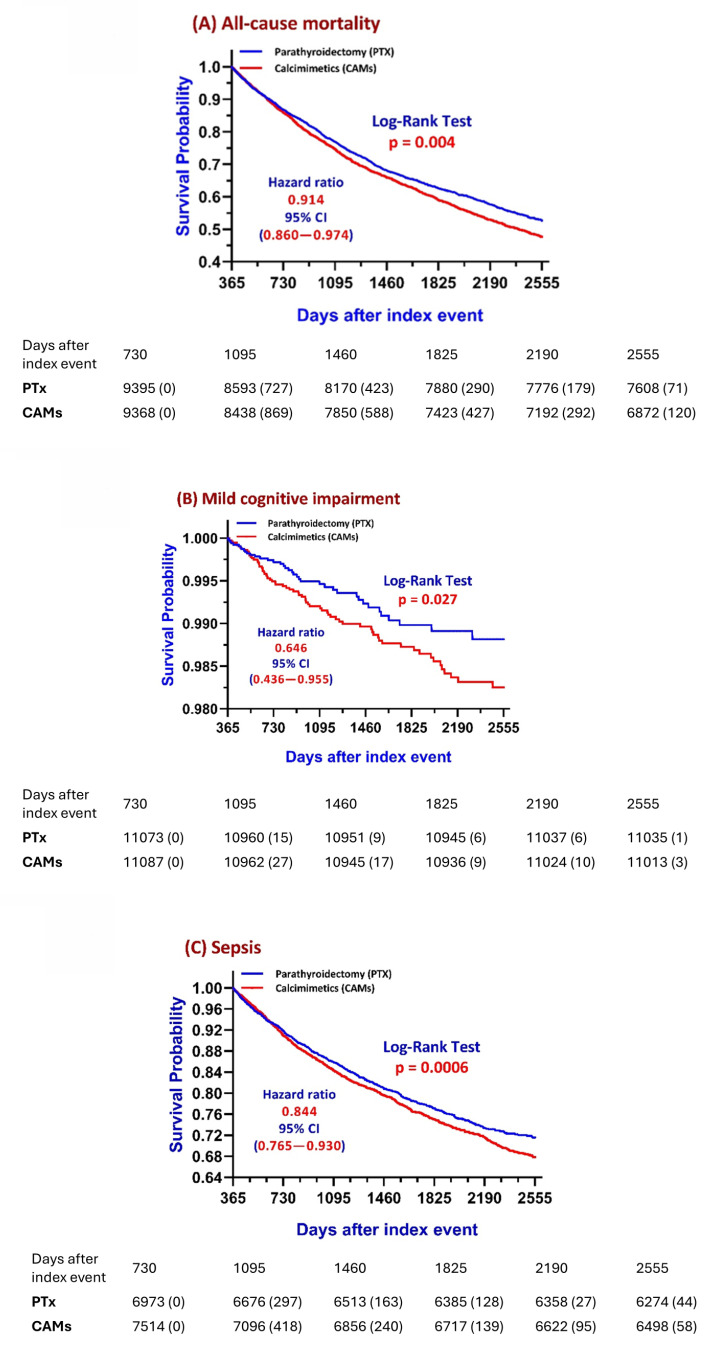
Kaplan-Meier survival curves for major outcomes after propensity score matching. (A) All-cause mortality: PTx was associated with lower mortality risk compared with CAMs (HR 0.91, 95% CI 0.86-0.97, p = 0.004). (B) Mild cognitive impairment (MCI): PTx was associated with lower incidence of MCI (HR 0.65, 95% CI 0.44-0.96, p = 0.027). (C) Sepsis: PTx was associated with lower sepsis risk compared with CAMs (HR 0.84, 95% CI 0.77-0.93, p < 0.001). Numbers at risk are shown below each panel. Group comparisons were assessed using log-rank tests; hazard ratios were estimated using Cox proportional hazards models.

**Figure 4:**
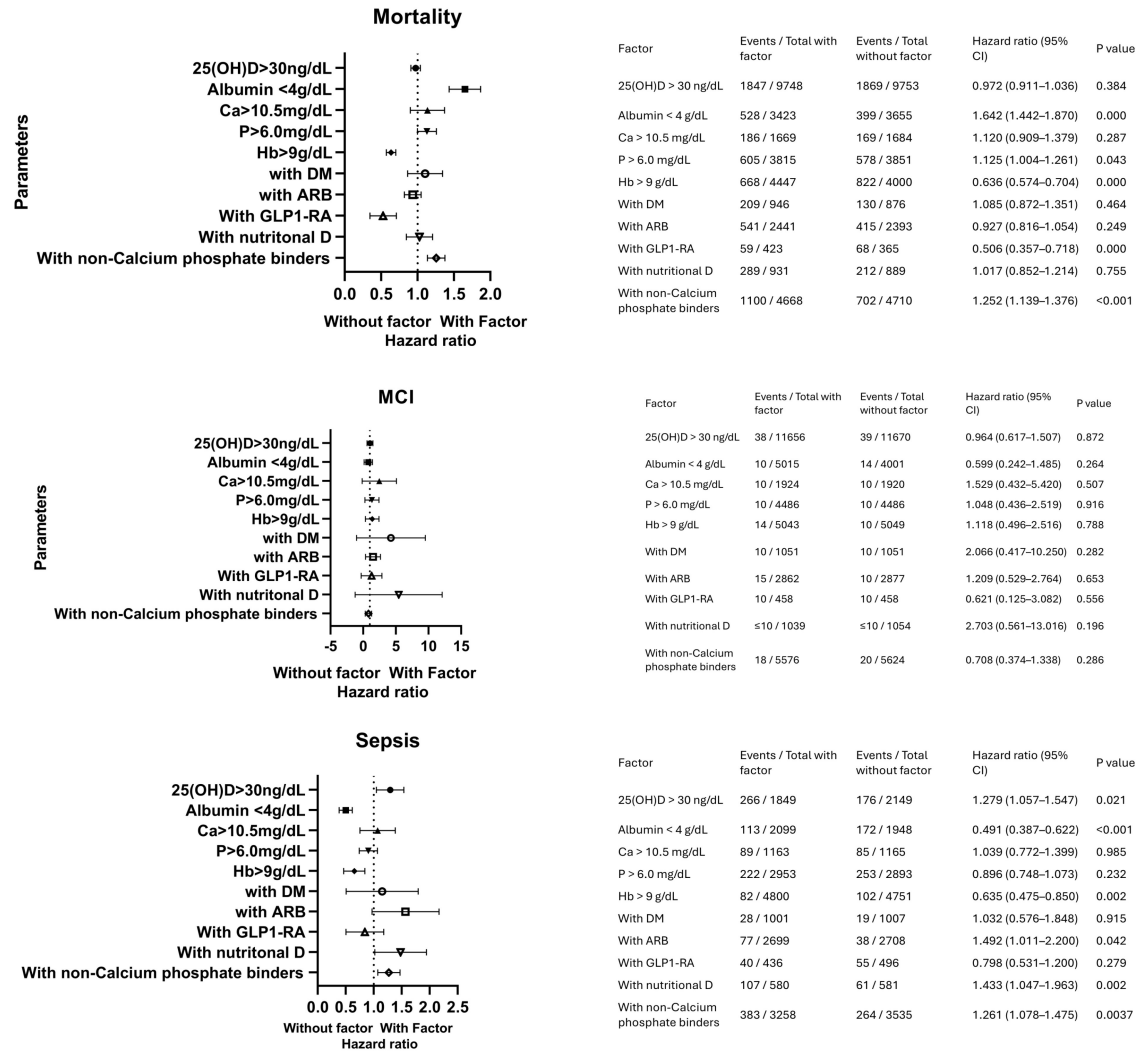
Subgroup analyses of treatment effects comparing PTx and CAMs. Forest plots show hazard ratios with 95% confidence intervals for prespecified clinical and biochemical subgroups. (A) Mortality: the association between PTx and lower mortality was generally consistent across subgroups; stronger associations were observed in patients with hemoglobin >9 g/dL, whereas higher risk was noted in those with low albumin (<4 g/dL), phosphorus >6.0 mg/dL, nutritional vitamin D use, and non-calcium phosphate binder therapy. (B) Mild cognitive impairment (MCI): no significant interaction was observed across subgroups. (C) Sepsis: PTx was associated with lower sepsis risk across most subgroups, whereas higher 25(OH)D levels, ARB use, nutritional vitamin D, and non-calcium phosphate binder therapy were associated with higher relative risk. Hazard ratios were estimated using Cox proportional hazards models.

PTx was associated with a significantly lower risk of all-cause mortality compared with CAM therapy. Kaplan-Meier curves showed early separation after the first year and persistent divergence throughout follow-up (Figure 3A). In Cox proportional hazards analysis, PTx was associated with an 8.6% lower mortality risk (HR 0.914, 95% CI 0.860-0.972, p = 0.004). Figure 4A illustrated the subgroup analysis among the subjects receiving PTx. Patients with adequate nutritional and hematologic profiles, particularly those with higher hemoglobin (> 9 g/dL) experienced significantly lower mortality risk, highlighting the protective effect of preserved nutritional status. Conversely, disturbances in mineral metabolism, such as hyperphosphatemia (> 6.0 mg/dL) or hypoalbuminemia was associated with greater risk, though the overall survival benefit of PTx remained consistent. Medication-related subgroups revealed modest risk increases among non-calcium phosphate binders (HR: 1.252, 95%CI:1.139-1.376, p < 0.05), whereas exposure to GLP1-RA had a protective impact (HR: 0.506, 95%CI:0.357-0.718, p < 0.05) (Figure 4A). To assess the robustness of the primary survival findings, additional landmark sensitivity analyses were performed using propensity score-matched cohorts with follow-up windows starting at 90 days and 180 days after the index date. Minor variations in cohort counts were observed across repeated analyses because TriNetX returns data dynamically according to contributing healthcare organizations at the time of query execution. In the 90-2100 day analysis, which included 760 patients in the PTx group and 28,372 in the CAMs group, PTx was associated with significantly lower mortality than CAMs (HR 0.451, 95% CI 0.357-0.570; log-rank P < 0.0001). Similar findings were observed in the 180-2100 day analysis, which included 754 patients in the PTx group and 28,492 in the CAMs group (HR 0.447, 95% CI 0.321-0.623; P = 0.0021).

### Outcomes Related to CKD-MBD (Fracture and Cardiovascular Events)

With respect to CKD-MBD-related outcomes, fracture risk did not significantly differ between groups (HR 1.090, 95% CI 0.956-1.242, p = 0.197). Likewise, no significant difference was observed for MACE (HR 0.857, 95% CI 0.811-1.129, p = 0.600) or hungry bone disease (HR 0.653, 95% CI 0.390-1.092, p = 0.468).

### Cognitive Impairment

No significant difference was observed in overall cognitive impairment between groups (HR 1.024, 95% CI 0.855-1.227, p = 0.795). However, PTx was associated with a significantly lower risk of mild cognitive impairment (MCI) compared with CAM therapy (HR 0.646, 95% CI 0.436-0.955, p = 0.027) (Figure 3B). Subgroup analysis did not identify any significant modifier of MCI risk, with all confidence intervals crossing unity (Figure 4B).

### Sepsis

PTx was also associated with a reduced risk of sepsis (HR 0.76, 95% CI 0.63-0.91, p = 0.004) (Figure 3C). In subgroup analyses, serum albumin level, hemoglobin level, nutritional vitamin D use, non-calcium phosphate binder therapy, and 25(OH)D status demonstrated significant associations with sepsis risk. Other factors, including calcium, phosphorus, diabetes, and newer antidiabetic agents, were not significantly associated with sepsis risk (Figure 4C).

## Discussion

In this large multicenter real-world cohort of patients with ESRD and severe SHPT, PTx was associated with significantly lower risks of all-cause mortality, mild cognitive impairment, and sepsis compared with CAM therapy. In contrast, fracture, MACE, hungry bone disease, overall cognitive impairment, and malignancy were comparable between groups.

### Mortality Benefit

The most consistent finding was the lower long-term mortality associated with PTx. Although the effect size was modest, survival curves showed early and persistent separation favoring surgery. This benefit may relate to durable PTH control, reduced calcium-phosphate burden, and mitigation of chronic inflammation. Residual mortality risk remained higher among patients with hypoalbuminemia, hyperphosphatemia, and those requiring non-calcium phosphate binders, indicating that malnutrition and severe mineral dysregulation continue to influence prognosis even after surgery. The consistency of mortality reduction across alternative landmark windows (90 and 180 days) further supports that the observed survival benefit of PTx was robust and unlikely to be explained solely by survivor bias related to delayed follow-up initiation. Previous studies have consistently demonstrated that parathyroidectomy (PTx) confers a survival advantage compared with standard care in patients with severe SHPT. A meta-analysis by Song *et al*. reported an association between PTx and improved survival 12, and similar findings have been observed in large real-world cohorts, including the Japanese dialysis registry 5. Calcimimetics (CAMs), on the other hand, effectively reduce parathyroid hormone levels through activation of the calcium-sensing receptor and may improve biochemical control without increasing calcium load, thereby potentially mitigating vascular calcification compared with active vitamin D therapy 13, 14. However, evidence regarding their impact on hard clinical outcomes remains heterogeneous. While individual agents such as etelcalcetide have demonstrated efficacy in PTH suppression and have shown favorable effects on surrogate markers—including modulation of calcium balance 15 , reduction in fibroblast growth factor 23 and attenuation of left ventricular hypertrophy 16 , and improvement in serum calcification propensity (T₅₀)^17^—these findings are primarily mechanistic or intermediate outcomes rather than definitive clinical endpoints. In contrast, comparative observational data suggest that PTx may provide more durable survival benefits than calcimimetic therapy in patients with advanced SHPT 7. The sustained and profound reduction in PTH achieved with surgery may contribute to improved outcomes through reduction of mineral imbalance, vascular calcification, and systemic inflammation. In our cohort, the mortality benefit associated with PTx remained evident across analyses, although its magnitude appeared influenced by underlying patient characteristics.

Subgroup analyses further highlighted the importance of nutritional and metabolic status. Patients with hypoalbuminemia exhibited less favorable outcomes, consistent with the established role of the malnutrition-inflammation complex in dialysis populations 18,19. Additionally, the observed associations between higher mortality risk and the use of non-calcium phosphate binders, angiotensin receptor blockers, and nutritional vitamin D should be interpreted with caution. These treatments are more commonly prescribed in patients with more severe disease, persistent biochemical abnormalities, or greater comorbidity burden, and thus likely reflect confounding by indication rather than direct adverse effects 20, 21. For instance, use of non-calcium phosphate binders often indicates difficult-to-control hyperphosphatemia 18, while nutritional vitamin D may be a marker of underlying metabolic instability. Taken together, these findings suggest that PTx is associated with a consistent survival advantage in patients with severe SHPT, but that this benefit is modulated by the severity of mineral metabolism disorders and the presence of coexisting comorbidities. Optimal long-term outcomes therefore require not only appropriate selection of surgical intervention, but also comprehensive management of phosphate balance, nutritional status, and concurrent therapies.

### Outcomes Related to CKD-MBD

Unlike several prior observational studies and recent meta-analyses, we did not observe a significant reduction in fracture risk after PTx, which contrasts with reports suggesting a protective skeletal effect of definitive SHPT treatment 8, 22. The EVOLVE trial also demonstrated that cinacalcet may reduce fracture events in older dialysis patients 6. while other studies have reported favorable changes in bone turnover markers after calcimimetic therapy 23. The discrepancy between our findings and prior literature may reflect differences in cohort composition, dialysis-related frailty, competing mortality, follow-up duration, patient selection, residual confounding, or limitations of administrative fracture coding. Although earlier studies emphasized greater fracture reduction in women and elderly populations, our real-world ESRD cohort suggests that skeletal outcomes are influenced by multiple factors beyond PTH control alone.

Similarly, hungry bone disease, a classic complication after PTx related to rapid skeletal remineralization, was not significantly different between groups in our study 24, 25, This finding is consistent with more recent cohort data suggesting improved perioperative management and modern calcium/vitamin D supplementation may attenuate the clinical burden of postoperative hungry bone disease 26. Our findings further suggest that with modern perioperative management and careful calcium/vitamin D supplementation, the clinical burden of hungry bone disease may be substantially reduced. For cardiovascular outcomes, we observed no significant difference in MACE between groups, consistent with prior reports that cardiovascular morbidity in ESRD is driven by multiple factors beyond PTH excess, including hypertension, diabetes, vascular calcification, and chronic inflammation. Although PTx provides clear biochemical control and may improve bone turnover, these benefits alone may be insufficient to alter the complex cardiovascular trajectory of dialysis patients. Taken together, our data indicate that PTx was associated with significant survival benefit, whereas its effects on fracture prevention and cardiovascular outcomes were less consistent in this real-world ESRD cohort than suggested by prior meta-analytic evidence.

### Neurocognitive Protection

An additional finding of our study is the potential neuroprotective role of PTx. Cognitive impairment is highly prevalent in dialysis patients and is driven by multifactorial mechanisms including uremic toxin accumulation, vascular calcification, oxidative stress, and dialysis-related cerebral hypoperfusion 27-29. Elevated PTH itself is increasingly recognized as an independent contributor to neurocognitive dysfunction, interfering with acetylcholine metabolism and synaptic transmission, and associating with structural brain changes 30, 31.

PTx was associated with a significantly lower risk of mild cognitive impairment (MCI), whereas no significant difference was observed for overall cognitive impairment or dementia. This finding contrasts with the clear survival and sepsis benefits observed with PTx, and likely reflects the multifactorial nature of neurodegeneration in end-stage renal disease (ESRD). Elevated parathyroid hormone (PTH) has been associated with cerebrovascular damage, endothelial dysfunction, and neuroinflammation, all of which contribute to white matter injury and accelerated cognitive decline in patients with advanced kidney disease 32, 33. In this context, PTx could theoretically mitigate cognitive decline by lowering PTH levels, reducing calcium-phosphate burden, and alleviating vascular calcification and oxidative stress. Our previous result also illustrated the association between hypoalbuminemia and the cognitive impairment in dialysis patients 34. However, the absence of a measurable effect on MCI risk in our cohort suggests that PTH reduction alone is insufficient to modify the complex interplay of uremic toxins, malnutrition, vascular injury, and amyloid dysregulation that drive neurodegeneration. A more integrated therapeutic strategy—combining PTx with optimized nutritional support, preservation of serum albumin, and interventions targeting uremic toxins—may therefore be necessary to achieve meaningful cognitive benefits in ESRD populations. Further studies should be initiated to elucidate the association of the neurodenerative disorder and secondary hyperparathyroidism.

### Infection and Immunoprotection

In patients who underwent parathyroidectomy, several factors demonstrated significant associations with sepsis risk. Albumin < 4 g/dL (HR 0.49, 95% CI 0.39-0.62, P < 0.001) and hemoglobin > 9 g/dL (HR 0.48, 95% CI 0.34-0.85, P = 0.002) were associated with lower observed sepsis risk. Conversely, 25(OH)D > 30 ng/mL (HR 1.55, 95% CI 1.06-1.28, P = 0.021), ARB use (HR 2.20, 95% CI 1.49-2.20, P = 0.042), nutritional vitamin D (HR 1.96, 95% CI 1.43-1.96, P = 0.002), and non-calcium phosphate binders (HR 1.48, 95% CI 1.26-1.48, P = 0.0037) were associated with higher sepsis risk.

These findings should be interpreted with caution. Although albumin <4 g/dL was associated with a lower observed sepsis risk, this result is inconsistent with established clinical evidence showing that hypoalbuminemia is strongly associated with infection-related morbidity and mortality in hemodialysis patients 35, 36. This discrepancy likely reflects residual confounding or selection bias rather than a true protective effect.

In contrast, the association between hemoglobin > 9 g/dL and lower sepsis risk is biologically plausible. Maintaining adequate hemoglobin levels has been associated with improved clinical outcomes 37, while lower hemoglobin levels are linked to increased hospitalization and mortality in dialysis patients 38. This is consistent with evidence suggesting that anemia correction may improve immune function, reduce oxidative stress, and decrease adverse outcomes in ESRD populations 39, 40. For patients undergoing PTx, adequate hemoglobin levels may therefore contribute to reduced susceptibility to infection.

In contrast, patients prescribed nutritional vitamin D or non-calcium phosphate binders showed higher sepsis risk. This likely reflects confounding by indication, as these therapies are more frequently used in patients with more severe mineral bone disorder or greater comorbidity burden. Vitamin D supplementation, although known to modulate innate immunity and induce antimicrobial peptides 41, has shown inconsistent effects on infection outcomes in CKD populations. Similarly, non-calcium phosphate binder use may identify patients with more advanced hyperphosphatemia and systemic vulnerability 42. Taken together, these findings suggest that hemoglobin status may be associated with sepsis risk in PTx patients, whereas therapies targeting mineral metabolism may serve as markers of disease severity rather than independent risk factors.

### Study Limitations

This study has several limitations. First, its retrospective design using an EMR-based database may introduce residual confounding despite rigorous propensity score matching. Certain unmeasured variables such as dialysis adequacy, nutritional status, inflammatory markers, and medication adherence were not available, which could influence outcomes. Second, diagnostic accuracy relied on ICD-10-CM coding, which may be subject to misclassification bias, particularly for cognitive impairment and sepsis. Third, laboratory data such as serum PTH trajectories, calcium-phosphate product, and vitamin D levels were incompletely available in the TriNetX dataset, limiting mechanistic insight. Finally, generalizability may be limited to healthcare systems represented in TriNetX, and unmeasured practice variation in surgical technique or CAM adherence could affect outcomes. Despite these limitations, the large sample size, robust matching, and consistent results across sensitivity and subgroup analyses strengthen the validity of our findings.

## Conclusion

Parathyroidectomy is associated with improved survival compared with calcimimetics in ESRD patients with severe SHPT. Importantly, PTx also reduces the risks of cognitive impairment and sepsis, outcomes that are critical to patient quality of life yet underappreciated in prior research. While CAMs remain valuable for patients unable to undergo surgery, these findings support broader consideration of PTx as a preferred therapeutic strategy in selected populations.

## Figures and Tables

**Table 1:** Coding of clinical indicators of the study

Death
Demographics: Deceased
Fracture (ICD-10-CM codes)
S12: Cervical vertebra / neck S22: Rib, sternum, thoracic spine S32: Lumbar spine, pelvis S42: Shoulder, upper arm S52: Forearm S62: Wrist, hand S72: Femur S82: Lower leg, ankle S92: Foot, toe
Major Adverse Cardiovascular Events (MACE) (ICD-10-CM codes)
I20-I25: Ischemic heart diseases I63: Cerebral infarction I50: Heart failure I70-I79: Peripheral arterial disease I49: Other cardiac arrhythmias
Hungry Bone Syndrome
E83.81: Hungry bone syndrome M89.30: Hypertrophy of bone, unspecified site
Cognitive Impairment / Dementia (ICD-10-CM codes)
F03: Unspecified dementia F02: Dementia in other diseases classified elsewhere F01: Vascular dementia G30: Alzheimer's disease
Mild Cognitive Impairment (MCI)
G31.84: Mild cognitive impairment of uncertain or unknown etiology
Parathyroid Hormone (PTH)
LOINC 2731-8: Parathyrin, intact [Mass/volume] in Serum or Plasma
Malignancies (exploratory outcome) (ICD-10-CM codes)
C00-C14: Lip, oral cavity, pharynx C15-C26: Digestive organs C30-C39: Respiratory / intrathoracic organs C50: Breast C64-C68: Urinary tract
Calcimimetics (CAMs)
RxNorm 407990: Cinacalcet RxNorm 1876119: Etelcalcetide HCPCS J0604: Cinacalcet, oral, 1 mg (ESRD on dialysis)
Parathyroidectomy (PTx)
ICD-10-PCS: 0GBR0ZZ, 0GBR3ZZ, 0GBR4ZZ (Excision) ICD-10-PCS: 0GTR0ZZ, 0GTR4ZZ (Resection) ICD-10-PCS: 0G5R0ZZ, 0G5R3ZZ, 0G5R4ZZ (Destruction) SNOMED: 53304009, 12330002, 63382008, 171995003, 171997006, 41237009 CPT: 60500, 60502, 60505, 1014226 ICD-10-CM: Z98.89 (Other postprocedural states), Z90.89 (Acquired absence of other organs)
Dialysis
CPT: 1012740 (Dialysis services and procedures) CPT: 90945 (Dialysis, non-hemodialysis, peritoneal, hemofiltration, CRRT) CPT: 1012757 (Miscellaneous dialysis services) ICD-9-CM: 39.95 (Hemodialysis) ESRD-related service codes: monthly (2-3 visits / ≥4 visits), per-day service for patients >20 years

**Table 2:** Demographic and baseline characteristics between PTx CAMS groups

Parameter	PTx (n=14,281)	CAMs (n=19,311)	P value	Std Diff	PTx (n=11,290)	CAMs (n=11,290)	P value	Std Diff
Demographics								
Age at index, mean ± SD	60.8 ± 15.1	57.6 ± 15.2	<0.001	0.212	59.6 ± 15.6	59.4 ± 14.4	0.406	0.011
Female, n (%)	6,188 (42.7)	8,578 (44.6)	<0.001	0.039	4,936 (43.7)	4,963 (44.0)	0.717	0.005
Male, n (%)	8,224 (56.7)	10,556 (54.9)	0.001	0.037	6,291 (55.7)	6,265 (55.5)	0.728	0.005
White, n (%)	7,476 (51.6)	6,580 (34.2)	<0.001	0.356	4,912 (43.5)	4,945 (43.8)	0.658	0.006
Black or African American, n (%)	4,946 (34.1)	8,533 (44.4)	<0.001	0.211	4,475 (39.6)	4,468 (39.6)	0.924	0.001
Asian, n (%)	726 (5.0)	1,083 (5.6)	0.012	0.028	628 (5.6)	647 (5.7)	0.584	0.007
Hispanic or Latino, n (%)	2,599 (17.9)	2,221 (11.6)	<0.001	0.181	1,570 (13.9)	1,568 (13.9)	0.969	0.001
Underlying illness								
Hypertensive diseases, n (%)	13,763 (94.9)	17,001 (88.4)	<0.001	0.237	10,579 (93.7)	10,623 (94.1)	0.221	0.016
Ischemic heart disease, n (%)	8,272 (57.1)	8,378 (43.6)	<0.001	0.272	5,899 (52.2)	5,935 (52.6)	0.631	0.006
Other heart disease, n (%)	11,256 (77.6)	12,505 (65.0)	<0.001	0.281	8,403 (74.4)	8,473 (75.0)	0.284	0.014
Cerebrovascular disease, n (%)	3,643 (25.1)	3,305 (17.2)	<0.001	0.195	2,465 (21.8)	2,478 (21.9)	0.834	0.003
Diseases of arteries/arterioles, n (%)	6,845 (47.2)	6,275 (32.6)	<0.001	0.301	4,691 (41.6)	4,689 (41.5)	0.978	<0.001
Diabetes mellitus, n (%)	10,080 (69.5)	10,219 (53.2)	<0.001	0.341	7,213 (63.9)	7,261 (64.3)	0.505	0.009
Medications								
Beta blockers, n (%)	11,353 (78.3)	14,134 (73.5)	<0.001	0.112	8,640 (76.5)	8,635 (76.5)	0.937	0.001
Antilipemic agents, n (%)	8,961 (61.8)	10,592 (55.1)	<0.001	0.137	6,572 (58.2)	6,925 (61.3)	<0.001	0.064
Antihypertensives, other, n (%)	8,723 (60.2)	9,748 (50.7)	<0.001	0.191	6,582 (58.3)	5,961 (52.8)	<0.001	0.111
ACE inhibitors, n (%)	3,150 (21.7)	4,837 (25.2)	<0.001	0.081	2,587 (22.9)	2,609 (23.1)	0.728	0.005
Angiotensin II inhibitors, n (%)	3,646 (25.2)	4,367 (22.7)	<0.001	0.057	2,763 (24.5)	2,685 (23.8)	0.225	0.016
Calcium channel blockers, n (%)	8,532 (58.9)	11,098 (57.7)	0.037	0.023	6,599 (58.4)	6,569 (58.2)	0.686	0.005
Insulin, n (%)	9,557 (65.9)	10,294 (53.5)	<0.001	0.255	6,917 (61.3)	6,891 (61.0)	0.723	0.005
Vitamin D, n (%)	7,051 (48.6)	9,421 (49.0)	0.508	0.007	5,465 (48.4)	5,488 (48.6)	0.759	0.004
Bisphosphonates, n (%)	100 (0.7)	114 (0.6)	0.268	0.012	75 (0.7)	75 (0.7)	1.000	<0.001
GLP-1 analogues, n (%)	393 (2.7)	304 (1.6)	<0.001	0.078	240 (2.1)	242 (2.1)	0.927	0.001
DPP-4 inhibitors, n (%)	659 (4.5)	523 (2.7)	<0.001	0.098	401 (3.6)	404 (3.6)	0.914	0.001
Calcium, n (%)	8,711 (60.1)	8,875 (46.2)	<0.001	0.282				

**Table 3:** Laboratory data between PTx CAMS groups

Parameter	PTx (n, %)	CAMs (n, %)	P value	Std Diff	PTx (n, %)	CAMs (n, %)	P value	Std Diff
	Before PSM				After PSM			
Phosphate (mg/dL)	4.6 ± 1.7	5.2 ± 1.9	< 0.001	0.307	4.7 ± 1.7	5.1 ± 1.8	< 0.001	0.237
3.5-5.5 mg/dL	8,873 (61.2)	10,638 (55.3)	< 0.001	0.119	6,668 (59.1)	6,557 (58.1)	0.134	0.020
5.5-6.5 mg/dL	5,886 (40.6)	7,912 (41.2)	0.308	0.011	4,405 (39.0)	4,783 (42.4)	< 0.001	0.068
6.5-7.5 mg/dL	4,027 (27.8)	5,964 (31.0)	< 0.001	0.071	3,025 (26.8)	3,558 (31.5)	< 0.001	0.104
2.5-3.5 mg/dL	5,811 (40.1)	5,751 (29.9)	< 0.001	0.214	4,254 (37.7)	3,692 (32.7)	< 0.001	0.104
Calcium (mg/dL)	8.8 ± 0.8	9.0 ± 0.9	< 0.001	0.208	8.8 ± 0.8	8.9 ± 0.9	< 0.001	0.069
8.5-10.5 mg/dL	11,682 (80.6)	14,992 (78.0)	< 0.001	0.064	8,936 (79.1)	8,889 (78.7)	0.443	0.010
10.5-12 mg/dL	1,590 (11.0)	2,980 (15.5)	< 0.001	0.134	1,392 (12.3)	1,437 (12.7)	0.366	0.012
12-14 mg/dL	141 (1.0)	224 (1.2)	0.091	0.019	111 (1.0)	125 (1.1)	0.360	0.012
7-8.5 mg/dL	9,085	9,719	< 0.001	0.246	6,513	6,533	0.788	0.004
Creatinine (mg/dL)	6.1 ± 3.2	7.9 ± 4.0	< 0.001	0.495	6.3 ± 3.3	7.6 ± 3.6	< 0.001	0.366
Hemoglobin (g/dL)	10.0 ± 1.9	10.3 ± 1.9	< 0.001	0.125	10.1 ± 2.0	10.2 ± 1.9	< 0.001	0.095
9-10 g/dL	9,212 (63.5)	10,397 (54.1)	< 0.001	0.193	6,704 (59.4)	6,710 (59.4)	0.935	0.001
10-11 g/dL	9,388 (64.8)	11,071 (57.6)	< 0.001	0.148	6,957 (61.6)	6,938 (61.5)	0.795	0.003
11-12 g/dL	8,359 (57.7)	9,969 (51.9)	< 0.001	0.117	6,192 (54.8)	6,213 (55.0)	0.779	0.004
>12 g/dL	6,436 (44.4)	7,796 (40.5)	< 0.001	0.078	4,818 (42.7)	4,817 (42.7)	0.989	< 0.001
Other labs								
Platelets (×10⁹/L)	205.1 ± 91.3 (12,552; 86.6)	203.1 ± 83.2 (16,539; 86.0)	0.050	0.023	205.3 ± 90.3 (9,594; 85.0)	201.3 ± 83.3 (9,931; 88.0)	0.002	0.045
Alkaline phosphatase (U/L)	121.8 ± 94.6	134.7 ± 114.1	< 0.001	0.123	121.5 ± 97.5	131.9 ± 98.6	< 0.001	0.106
Albumin (g/dL)	3.3 ± 0.7	3.5 ± 0.7	< 0.001	0.309	3.3 ± 0.7	3.5 ± 0.7	< 0.001	0.202
3.5-4.0 g/dL	7,752 (53.5)	9,796 (51.0)	< 0.001	0.050	5,797 (51.3)	5,962 (52.8)	0.028	0.029
4.0-4.5 g/dL	5,074 (35.0)	7,133 (37.1)	< 0.001	0.044	3,876 (34.3)	4,175 (37.0)	< 0.001	0.055
Cholesterol (mg/dL)	141.7 ± 47.6	149.1 ± 47.9	< 0.001	0.156	144.2 ± 47.8	145.8 ± 47.6	0.124	0.033
LDL cholesterol (mg/dL)	72.6 ± 36.9	77.0 ± 37.1	< 0.001	0.118	74.4 ± 37.1	74.6 ± 36.9	0.785	0.006
HDL cholesterol (mg/dL)	42.5 ± 17.8	43.7 ± 17.1	< 0.001	0.069	43.3 ± 18.2	43.1 ± 17.0	0.570	0.012
BNP (pg/mL)	1,670.4 ± 4,096.1 (4,178; 28.8)	2,065.4 ± 7,829.1 (4,261; 22.2)	0.004	0.063	1,570.4 ± 3,769.9 (3,021; 26.8)	2,168.6 ± 7,385.7 (2,791; 24.7)	< 0.001	0.102
HbA1c (%)	6.4 ± 1.7	6.2 ± 1.7	< 0.001	0.136	6.4 ± 1.7	6.3 ± 1.7	0.007	0.050
LVEF (%)	52.9 ± 14.6	54.1 ± 14.3	0.023	0.079	53.1 ± 14.7	53.2 ± 14.6	0.860	0.007
25(OH)D (ng/mL)	27.9 ± 17.4	26.0 ± 16.4	0.001	0.113	27.3 ± 16.9	26.7 ± 16.5	0.348	0.039

## Data Availability

The datasets analyzed during the current study were obtained from the TriNetX research network, which provides access to de-identified patient-level data from participating healthcare organizations. Due to data use agreements and privacy regulations, the raw data cannot be shared publicly. Access to TriNetX data is available upon reasonable request to TriNetX (https://trinetx.com) for researchers with an institutional license.

## Abbreviations

ESRD: End-stage renal disease
SHPT: Secondary hyperparathyroidism
PTx: Parathyroidectomy
CAMs: Calcimimetics
PTH: Parathyroid hormone
MACE: Major adverse cardiovascular events
MCI: Mild cognitive impairment
HR: Hazard ratio
CI: Confidence interval
